# Association Between Amino Acid Levels and Thyroid Function Among Iranian Adults: A Cross‐Sectional Analysis of Bushehr Elderly Health (BEH) Program

**DOI:** 10.1002/edm2.70375

**Published:** 2026-11-03

**Authors:** Mohamad Mehdi Khadembashiri, Kazem Khalagi, Shahrzad Mohseni, Alireza Amanollahi, Fatemeh Bandarian, Noushin Fahimfar, Iraj Nabipour, Mahnaz Pejman Sani, Farideh Razi

**Affiliations:** ^1^ Metabolomics and Genomics Research Center Endocrinology and Metabolism Molecular‐Cellular Sciences Institute, Tehran University of Medical Sciences Tehran Iran; ^2^ Osteoporosis Research Center Endocrinology and Metabolism Clinical Sciences Institute, Tehran University of Medical Sciences Tehran Iran; ^3^ Obesity and Eating Habits Research Center Endocrinology and Metabolism Clinical Sciences Institute, Tehran University of Medical Sciences Tehran Iran; ^4^ Endocrinology and Metabolism Research Center Endocrinology and Metabolism Clinical Sciences Institute, Tehran University of Medical Sciences Tehran Iran; ^5^ Trauma and Injury Research Center Iran University of Medical Sciences Tehran Iran; ^6^ Department of Epidemiology and Biostatistics, School of Public Health Tehran University of Medical Sciences Tehran Iran; ^7^ The Persian Gulf Marine Biotechnology Research Center The Persian Gulf Biomedical Sciences Research Institute, Bushehr University of Medical Sciences Bushehr Iran; ^8^ Department of Endocrinology, Shariati Hospital, School of Medicine Tehran University of Medical Sciences Tehran Iran

**Keywords:** amino acid, metabolomics, thyroid

## Abstract

**Introduction:**

Thyroid hormones regulate key metabolic processes; however, the association between thyroid function and amino acid metabolism varies across studies. This study aimed to investigate the relationship between thyroid biomarkers and circulating amino acid concentrations in adults aged 50 years and older.

**Methods:**

We initially enrolled a random subsample of 824 adults aged 50 years and older from the second recruitment of the Bushehr Elderly Health (BEH) Program. Because the clinical and subclinical hyperthyroidism groups were very small, 14 participants from these groups were excluded from the primary between‐group and regression analyses. Therefore, the primary analytical sample comprised 810 participants with euthyroid status, subclinical hypothyroidism, or clinical hypothyroidism. Associations between amino acid concentrations and thyroid biomarkers were assessed using multivariable linear regression models.

**Results:**

The initial sample included 335 men and 489 women, with mean ages of 62.27 ± 8.57 and 61.47 ± 7.52 years, respectively. Although several nominal associations were observed between amino acids and TSH in crude or adjusted analyses, none remained statistically significant after false discovery rate (FDR) correction. No statistically significant differences in amino acid concentrations were observed between the thyroid disorder groups after FDR correction.

**Conclusion:**

No statistically significant associations between amino acid concentrations and thyroid biomarkers remained after correction for multiple comparisons. Likewise, no significant differences in amino acid concentrations were observed between the examined thyroid disorder groups after FDR correction. Larger longitudinal studies are needed to determine whether the nominal findings observed in the present study are reproducible and biologically meaningful.

## Graphical abstract

In Iranian adults aged ≥ 50 years, no statistically significant differences in amino acid concentrations or robust associations with thyroid biomarkers remained after false discovery rate correction. Nominal findings were exploratory and require confirmation in larger longitudinal studies.

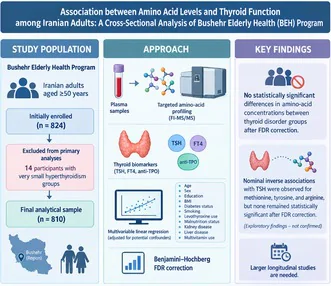

**Abbreviations**
ALP: alkaline phosphatase
ALT: alanine transaminase
AST: aspartate aminotransferase
BMI: body mass index
BMR: basal metabolic rate
C‐HyperT: clinical hyperthyroidism
C‐HypoT: clinical hypothyroidism
Cr: creatinine
DBP: diastolic blood pressure
DXA: dual‐energy x‐ray absorptiometry
ECL: electrochemiluminescence
EMRI: endocrinology and metabolism research institute
FDR: false discovery rate
HDL‐C: high‐densitylipoprotein‐cholesterol
LDL‐C: low‐density lipoprotein‐cholesterol
NMR: nuclear magnetic resonance
PGTMRC: persian gulf marine biotechnology research centre
SBP: systolic blood pressure
SC‐HyperT: subclinical hyperthyroidism
SC‐HypoT: subclinical hypothyroidism
T3: triiodothyronine
T4: thyroxine
TPO: thyroid peroxidase
TUMS: tehran university of medical sciences

## 1 Introduction

Thyroid dysfunction is common and is associated with substantial metabolic, cardiovascular and quality of life consequences [1, 2, 3]. Clinical hypothyroidism (C‐HypoT) is characterized by an elevated thyroid‐stimulating hormone (TSH) above the reference range accompanied by reduced free thyroxine (FT4), whereas subclinical hypothyroidism (SC‐HypoT) comprises elevated TSH with normal free thyroid hormone levels [4, 5]. Conversely, hyperthyroidism represents the opposite biochemical pattern, with suppressed TSH and either increased FT4 in clinical hyperthyroidism (C‐HyperT) or preserved FT4 in the subclinical form (SC‐HyperT) [6]. Globally, overt hypothyroidism is estimated to affect roughly 0.3%–5% of the general population, while subclinical hypothyroidism may be present in about 4%–12% (and higher in older adults), depending on the population and diagnostic thresholds used [7, 8]. Although clinical and subclinical hyperthyroidism occur less frequently, both remain clinically important because they are associated with adverse cardiovascular and metabolic outcomes [9].

Rising life expectancy has increased the proportion of older adults worldwide, underscoring the health implications of aging [10, 11]. Aging leads to hormonal and metabolic changes, particularly affecting thyroid function [12]. Aging is accompanied by changes in thyroid physiology, including a tendency toward higher TSH concentrations, altered turnover of T4 and T3, and a greater prevalence of thyroid autoantibodies [13, 14, 15]. Thyroid hormones are crucial for systemic metabolism, as they regulate energy balance, lipid and carbohydrate metabolism, protein turnover, thermogenesis, and growth and development [16]. Accordingly, excess thyroid hormone may promote a hypermetabolic state with increased energy expenditure and protein catabolism, whereas reduced thyroid hormone activity is generally associated with lower metabolic rate and slower substrate turnover [17, 18, 19]. At the cellular level, thyroid hormones act on virtually all tissues, modifying mitochondrial biogenesis and uncoupling, increasing Na+/K + ‐ATPase activity, and influencing the expression of genes involved in metabolic pathways, thereby altering global metabolic fluxes [18, 20, 21]. Recent metabolomics studies have shown that altered thyroid hormone status is associated with significant changes in circulating amino acid levels [22, 23]. Amino acids have roles beyond protein synthesis, including participation in energy production, nitrogen balance, biosynthetic pathways, redox regulation, and cellular signalling [24]. Therefore, amino acid profiling can serve as a sensitive indicator of metabolic state and a potential tool in diagnosing, monitoring, or better understanding thyroid disorders. While existing evidence highlights an association between thyroid function (levels of thyroid hormones) and amino acid levels, the findings are inconsistent [23, 25, 26, 27, 28, 29]. Prior metabolomic studies of thyroid disorders have been limited by small sample sizes, heterogeneous age ranges, non‐targeted platforms with limited quantitative precision, and inconsistent adjustment for confounders, which likely explains the conflicting associations reported to date.

Given the significant metabolic implications of thyroid disorders, the impact of aging on thyroid and metabolic function, and the inconsistent existing evidence, this study examined the relationship between thyroid biomarkers and plasma amino acid concentrations in Iranian adults aged 50 years and older participating in the Bushehr Elderly Health (BEH) Program. By applying a targeted, quantitative FI‐MS/MS amino acid panel to a relatively large age‐defined population with systematic adjustment for key confounders, this study aims to provide more precise and reproducible estimates of the thyroid–amino acid relationship.

## 2 Materials and Methods

### 2.1 Study Design and Participants

This cross‐sectional analysis used baseline data from the second recruitment of the Bushehr Elderly Health (BEH) Program, a prospective cohort study conducted by the Endocrinology and Metabolism Research Institute (EMRI) at Tehran University of Medical Sciences (TUMS) and Persian Gulf Marine Biotechnology Research Centre (PGTMRC) at Bushehr University of Medical Sciences to investigate the prevalence of non‐communicable diseases and their associated risk factors in an elderly population. In the first recruitment phase of the BEH study, 3000 individuals aged 60 years and older were enrolled. The second recruitment phase included 2000 adults aged 50 years and older. The detailed protocol and methodology of the study have been outlined previously [30, 31, 32]. This study was conducted according to the STROBE guidelines [33]. The study protocol was approved by the Ethics Committee of the Endocrinology and Metabolism Research Institute, Tehran University of Medical Sciences (IR.TUMS.EMRI.REC.1403.034). Written informed consent was obtained from all the participants. All research procedures adhered to the Declaration of Helsinki and its subsequent amendments [34].

After calculating the sample size, a random subsample of 824 participants aged 50 years and over from the 2000 participants in the second recruitment of the BEH Program were enrolled in the study. Because the hyperthyroid subgroups were too small to yield stable estimates, the 14 participants with clinical or subclinical hyperthyroidism were excluded from the primary group comparisons and regression models. Therefore, the primary analytical sample included 810 participants. Figure 1 illustrates the overview of the study population. Comprehensive data collection encompassed demographic, clinical, laboratory, dual‐energy X‐ray absorptiometry (DXA), and metabolite information [31, 32, 35].

**FIGURE 1:**
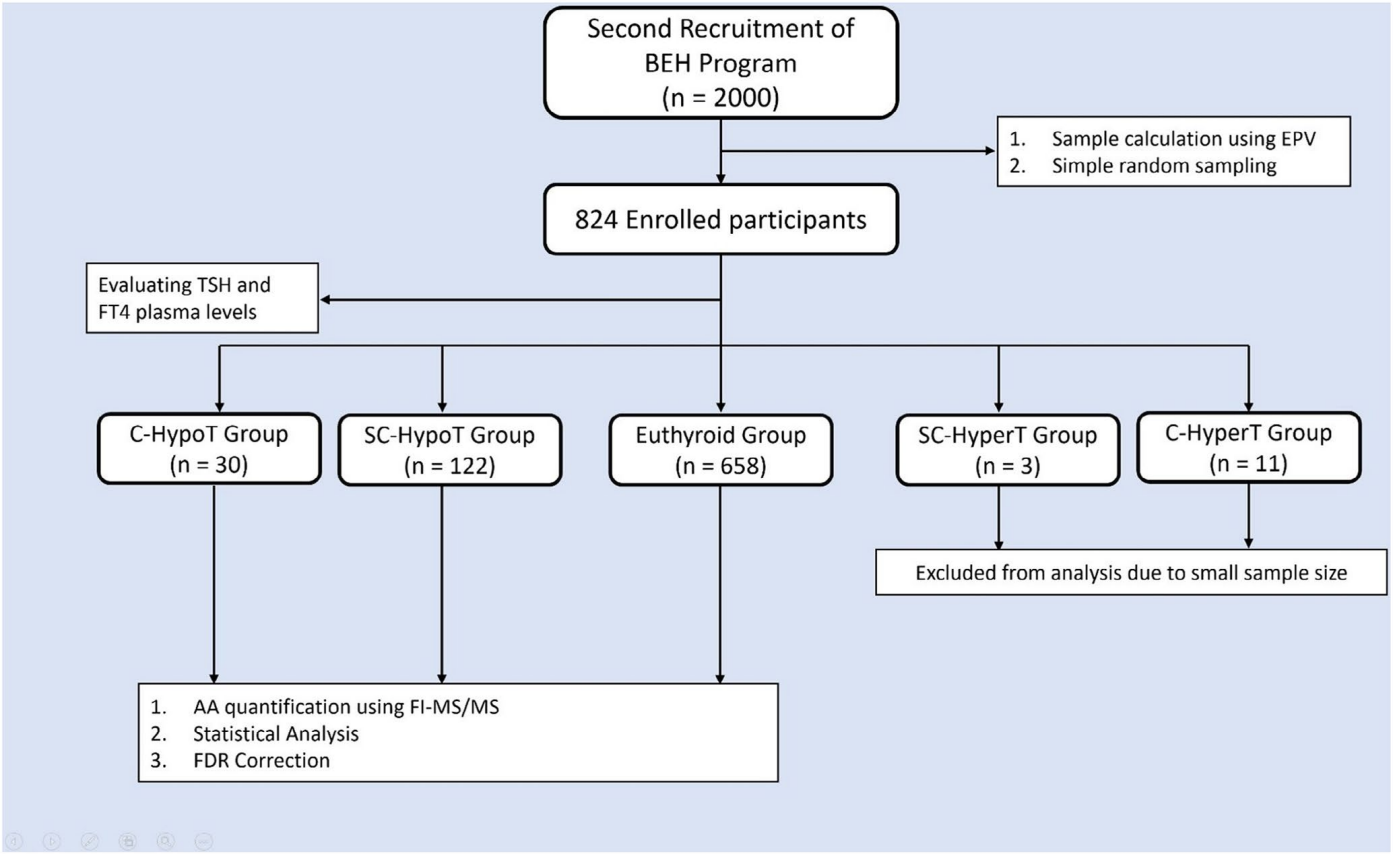
Study population overview. BEH, Bushehr elderly health; EPV, Events per predictor variable; TSH, Thyroid stimulating hormone; FT4, Free thyroxine; C‐HypoT, Clinical hypothyroidism; SC‐HypoT, Subclinical hypothyroidism; SC‐HyperT, Subclinical hyperthyroidism; C‐HyperT, Clinical hyperthyroidism; AA, Amino acid; FI‐MS/MS, Flow‐injection tandem mass spectrometry; FDR, False Discovery Rate.

### 2.2 Data Collection and Laboratory Measurements

Information on age, sex, educational attainment, smoking, medical history, and medication use was obtained through standardized interviews. Height, body weight, and systolic (SBP) and diastolic (DBP) blood pressure were measured during the clinical examination by trained personnel. Height and weight were measured using a fixed stadiometer and a digital scale, with participants wearing minimal clothing. Following a 15‐min rest period, SBP and DBP were measured twice on the right arm at 10‐min intervals, and the average of the two measurements was recorded. Body mass index (BMI) was calculated as weight in kilograms divided by height in meters squared.

Morning venous blood samples were collected following an overnight fast of 8–12 h. The comparative biochemical assessment included the quantification of total cholesterol, high‐density lipoprotein cholesterol (HDL‐C), low‐density lipoprotein cholesterol (LDL‐C), triglycerides, blood urea nitrogen, creatinine (Cr), aspartate aminotransferase (AST), alanine transaminase (ALT), and alkaline phosphatase (ALP) using commercial kits (Pars Azmun, Tehran, Iran). Lipid measurements were carried out using the CHOD‐PAP enzymatic colorimetric method for total cholesterol, the CHE&CHO method for LDL‐C and HDL‐C, and the GPO‐PAP method for triglycerides. The measurement method for the other markers are described elsewhere [30, 32].

TSH, FT4, and anti‐thyroid peroxidase (anti‐TPO) concentrations were measured using electrochemiluminescence immunoassays (ECL) with Roche Diagnostics reagents on a Cobas e 411 analyser (Roche Diagnostics GmbH, Mannheim, Germany). The corresponding catalogue numbers were 08429324190 for TSH, 09007741190 for FT4, and 07026935190 for anti‐TPO.

### 2.3 Amino Acid Analysis Using FI‐MS/MS

Circulating amino acids were quantified using a targeted flow‐injection tandem mass spectrometry (FI‐MS/MS) procedure. Plasma samples were prepared with appropriate internal standards and subjected to derivatization with acetyl chloride in 1‐butanol before instrumental analysis. The processed samples were introduced through an automated sampling system for mass‐spectrometric measurement.

Analyses were performed using a Thermo Scientific Dionex UltiMate 3000 HPLC system equipped with a binary pump and operated with the analytical column bypassed. The system was coupled to a SCIEX API 320 quadrupole mass spectrometer equipped with an electrospray ionization source. Amino acid concentrations were determined using calibration curves generated from the ratios of analyte peak areas to those of the corresponding internal standards. Quality‐control samples were analysed alongside study samples, and concentrations were calculated using the established calibration procedures. Quantification was based on calibration curves constructed from the ratios of metabolite peak areas to internal standards, and concentrations in study materials and QC samples were calculated accordingly. Comprehensive validation of the analytical method has been reported previously [36].

### 2.4 Definition of Terms

Diabetes was defined according to any of the following criteria: fasting plasma glucose ≥ 126 mg/dL, HbA1c ≥ 6.5%, or current use of antidiabetic medication. Current smoking was defined as daily or occasional use of cigarettes, hookah, or pipe.

Thyroid function categories were assigned according to the laboratory reference ranges for TSH and FT4. Participants were considered euthyroid when TSH was 0.27–4.2 mIU/mL and FT4 was 0.93–1.7 ng/dL. Clinical hypothyroidism (C‐HypoT) was defined as TSH > 4.2 mIU/mL with FT4 < 0.93 ng/dL, whereas subclinical hypothyroidism (SC‐HypoT) was defined as TSH > 4.2 mIU/mL with FT4 within the reference range. Clinical hyperthyroidism (C‐HyperT) was defined as TSH < 0.27 mIU/mL with FT4 > 1.7 ng/dL, and subclinical hyperthyroidism (SC‐HyperT) as TSH < 0.27 mIU/mL with FT4 within the reference range.

### 2.5 Statistical Analysis

Sample size adequacy was assessed using the number of subjects per variable (SPV) approach, based on the number of independent variables included in the multivariable models [37]. Continuous variables were summarized using means and standard deviations, and categorical variables were described as frequencies and percentages. The assumptions underlying the statistical tests for mean comparison (independent *t*‐test, one‐way ANOVA) and predictor–outcome were assessed. The predictors were linearly related to the outcomes, and the mean and standard deviation of the residuals of the regression model were zero and close to one (0.97–0.99), respectively. Multicollinearity was not observed in all multiple regression models greater than two. Multicollinearity was not observed in all multiple regression models greater than two. Univariate analyses were conducted using the independent *t*‐test, one‐way ANOVA, chi‐square, and Fisher's exact test. In the regression models, the amino acids were converted to *Z*‐scores. Linear regression was applied to examine the association between metabolites and thyroid hormone levels. To account for potential confounders (age, sex, education, BMI, diabetes, smoking, levothyroxine use, malnutrition status, kidney disease, liver disease and multivitamin use), multiple linear regression was employed. All *p*‐values were adjusted for multiple comparisons using the Benjamini–Hochberg procedure to control the false discovery rate (FDR). Analyses were performed using SPSS, Stata, and R, with a significance level set at 0.05. Heatmap plots were drawn with the ggplot2 package in R software.

## 3 Results

### 3.1 Baseline Characteristics of Participants

The initial study sample included 824 participants, comprising 335 men and 489 women. Mean age was 62.27 ± 8.57 years among men and 61.47 ± 7.52 years among women. The distribution of thyroid‐function categories was 658 euthyroid participants (79.8%), 122 with subclinical hypothyroidism (14.8%), 30 with clinical hypothyroidism (3.9%), 11 with clinical hyperthyroidism (1.2%), and 3 with subclinical hyperthyroidism (0.3%). Demographic and laboratory characteristics of participants are summarized in Table 1.

**TABLE 1:** Baseline characteristics of participants based on thyroid disorder status.

Parameters	Total (*n* = 824)	Euthyroid (*n* = 658)	Subclinical hypothyroidism (*n* = 122)	Clinical hypothyroidism (*n* = 30)	Subclinical hyperthyroidism (*n* = 3)	Clinical hyperthyroidism (*n* = 11)	*p* *
Age [mean ± standard deviation]	61.79 ± 7.96	61.57 ± 7.90	61.61 ± 8.07	65.05 ± 7.36	66.62 ± 12.27	67.13 ± 8.57	0.062
Sex [No. (%)]							**0.015**
Male	335 (40.7)	285 (43.3)	36 (29.5)	11 (36.7)	0 (0.0)	3 (27.3)	
Female	489 (59.3)	373 (56.7)	86 (70.5)	19 (63.3)	3 (100.0)	8 (72.7)	
BMI [mean ± standard deviation]	28.30 ± 5.01	28.05 ± 4.87	29.67 ± 5.83	28.50 ± 3.76	26.75 ± 7.80	27.58 ± 3.33	**0.004**
Smoke [no. (%)]							0.896
Yes	188 (22.8)	153 (23.3)	27 (22.1)	6 (20.0)	0 (0.0)	3 (27.3)	
No	636 (77.2)	505 (76.3)	95 (77.9)	24 (80.0)	3 (100.0)	8 (72.7)	
Education [no. (%)]							**0.013**
Illiterate	96 (11.7)	77 (11.7)	11 (9.0)	7 (23.3)	0 (0.0)	1 (9.1)	
Primary school	268 (32.5)	215 (32.7)	47 (38.5)	4 (13.3)	0 (0.0)	2 (18.2)	
Secondary school	270 (32.8)	227 (34.5)	31 (25.4)	8 (26.7)	0 (0.0)	4 (36.4)	
Academic	190 (23.1)	139 (21.1)	33 (27.0)	11 (36.7)	3 (100.0)	4 (36.4)	
Diabetes mellitus [No. (%)]							0.556
Yes	293 (35.8)	240 (36.6)	38 (31.7)	10 (33.3)	2 (66.7)	3 (27.3)	
No	526 (64.2)	415 (63.4)	82 (68.3)	20 (66.7)	1 (33.3)	8 (72.7)	
Kidney disease							0.473^&^
Yes	28 (3.4)	20 (3.0)	6 (4.9)	1 (3.3)	0 (0.0)	1 (9.1)	
No	795 (96.6)	637 (97.0)	116 (95.1)	29 (96.7)	3 (100.0)	10 (90.9)	
Liver diseases							0.573^&^
Yes	40 (4.9)	34 (5.2)	4 (3.3)	2 (6.7)	0 (0.0)	0 (0.0)	
No	783 (95.1)	623 (94.8)	118 (96.7)	28 (93.3)	3 (100.0)	11 (100.0)	
Levothyroxine use							< 0.001
Yes	118 (14.3)	69 (10.5)	30 (24.6)	7 (23.3)	3 (100.0)	9 (81.8)	
No	706 (85.7)	589 (89.5)	92 (75.4)	23 (76.7)	0 (0.0)	2 (18.2)	
Multivitamin use							0.801^&^
Yes	47 (5.7)	36 (5.5)	8 (6.6)	1 (3.3)	0 (0.0)	2 (18.2)	
No	777 (94.3)	622 (94.5)	114 (93.4)	29 (96.7)	3 (100.0)	9 (81.8)	
Malnutrition							0.345^&^
Yes	28 (3.4)	22 (3.3)	3 (2.5)	2 (6.7)	0 (0.0)	1 (9.1)	
No	796 (96.6)	636 (96.7)	119 (97.5)	28 (93.3)	3 (100.0)	10 (90.9)	
SBP [mean ± standard deviation]	131.11 ± 19.76	131.26 ± 19.56	129.76 ± 21.60	135.43 ± 17.13	128.33 ± 24.54	125.27 ± 15.48	0.362
DBP [mean ± standard deviation]	80.25 ± 10.52	80.20 ± 10.37	80.16 ± 11.37	83.33 ± 10.59	70.34 ± 14.50	78.18 ± 5.51	0.278
TSH (mIU/mL) [mean ± standard deviation]	3.65 ± 6.77	2.23 ± 0.95	6.27 ± 2.79	25.61 ± 25.88	0.13 ± 0.06	0.11 ± 0.03	**0.000**
FT4 (ng/dL) [mean ± standard deviation]	1.26 ± 0.25	1.28 ± 0.20	1.22 ± 0.17	0.73 ± 0.24	1.11 ± 0.17	2.26 ± 0.50	**0.000**
Anti TPO (IU/mL) [mean ± standard deviation]	66.34 ± 117.43	46.07 ± 81.74	126.08 ± 117.36	231.88 ± 192.34	44.96 ± 43.92	168.40 ± 207.11	**0.000**
Cholesterol (mg/dL) [mean ± standard deviation]	189.63 ± 42.59	189.21 ± 42.63	189.17 ± 40.85	211.27 ± 44.52	189.33 ± 42.10	161.27 ± 35.98	**0.020**
LDL‐C (mg/dL) [mean ± standard deviation]	110.08 ± 37.17	109.62 ± 37.15	109.97 ± 36.15	128.24 ± 40.97	105.13 ± 35.62	90.24 ± 25.84	**0.027**
HDL‐C (mg/dL) [mean ± standard deviation]	48.79 ± 11.19	48.78 ± 11.02	49.37 ± 11.25	45.09 ± 11.94	52.53 ± 11.58	52.34 ± 16.79	0.161
Triglyceride (mg/dL) [mean ± standard deviation]	152.68 ± 87.55	152.16 ± 85.23	151.59 ± 90.93	189.67 ± 116.67	158.33 ± 89.01	93.45 ± 39.49	0.069
Dyslipidemia [no. (%)]							**0.015**
Yes	602 (73.1)	472 (71.7)	95 (77.9)	28 (93.3)	2 (66.7)	6 (54.5)	
No	222 (26.9)	186 (28.3)	27 (22.1)	2 (6.7)	1 (33.3)	5 (45.5)	
Urea (mg/dL) [mean ± standard deviation]	31.28 ± 10.46	31.49 ± 10.86	30.07 ± 8.62	30.63 ± 9.36	36.0 ± 4.36	33.01 ± 8.72	0.370
Cr (mg/dL) [mean ± standard deviation]	1.07 ± 0.35	1.08 ± 0.37	1.05 ± 0.25	1.09 ± 0.22	0.95 ± 0.15	1.06 ± 0.34	0.668
ALT (IU/L) [mean ± standard deviation]	19.48 ± 9.69	19.50 ± 9.79	19.0 ± 8.26	20.50 ± 13.58	15.33 ± 3.05	21.64 ± 7.21	0.731
AST (IU/L) [mean ± standard deviation]	21.52 ± 7.63	21.31 ± 7.26	21.37 ± 7.04	26.80 ± 14.30	17.66 ± 1.52	22.18 ± 6.36	**0.000**
ALP (IU/L) [mean ± standard deviation]	227.48 ± 62.14	227.33 ± 60.06	229.20 ± 73.32	231.33 ± 64.31	210.67 ± 37.21	211.20 ± 54.32	0.907
FBS (mg/dL) [mean ± standard deviation]	111.75 ± 44.12	111.73 ± 44.15	112.25 ± 44.30	115.97 ± 50.42	98.33 ± 1.58	98.64 ± 26.22	0.877
HbA1C (%) [mean ± standard deviation]	6.35 ± 1.52	6.35 ± 1.50	6.39 ± 1.62	6.16 ± 1.33	6.57 ± 0.53	6.23 ± 1.45	0.754

*Note:* Statistically significant results (p < 0.05) are shown in bold.

Abbreviations: ALP, alkaline phosphatase; ALT, alanine transaminase; AST, aspartate aminotransferase; BMI, body mass index; Cr, creatinine; DBP, diastolic blood pressure; FT4, free thyroxine; HDL‐C, high‐density lipoprotein‐cholesterol; LDL‐C, low‐density lipoprotein‐cholesterol; SBP, systolic blood pressure; TPO, thyroid peroxidase; TSH, thyroid stimulating hormone.

* Continuous variables were compared using one‐way ANOVA and are presented as mean ± SD; categorical variables were compared using chi‐square, and Fisher's‐exact tests (&) and are presented as *n* (%). Comparisons were made across Euthyroid, Subclinical Hypothyroidism (SC‐HypoT), and Clinical Hypothyroidism (C‐HypoT) groups; Clinical and Subclinical Hyperthyroidism groups were excluded due to small sample size.

Mean TSH levels were markedly altered in C‐HypoT (25.61 ± 25.88) and C‐HyperT (0.11 ± 0.03) compared with the euthyroid group, and corresponding mean FT4 levels were 0.73 ± 0.24 and 2.26 ± 0.50, respectively. For anti‐TPO, the highest value was observed in the C‐HypoT group (231.88 ± 192.34). BMI varied significantly among the different disease groups, with SC‐HypoT showing the highest mean at 29.67 ± 5.83. Levels of LDL‐C (*p* ≤ 0.017), total cholesterol (*p* ≤ 0.01), and AST (*p* ≤ 0.003) were increased in the C‐HypoT group compared with both SC‐HypoT and euthyroid individuals.

Among the thyroid biomarkers assessed, only anti‐TPO concentrations differed significantly between men and women (*p* = 0.009). Sex‐related differences were also observed for total cholesterol, HDL cholesterol, and LDL cholesterol, but not for triglycerides (*p* ≤ 0.008). Several renal and hepatic markers, including urea, creatinine, AST, ALT and ALP, also differed significantly by sex (*p* ≤ 0.01). Detailed sex‐stratified characteristics are provided in Table S1.

### 3.2 Serum Level of Amino Acids in Different Thyroid Disorders

Given the very small number of participants with clinical hyperthyroidism (*n* = 11) and subclinical hyperthyroidism (*n* = 3), these groups were excluded from between‐group comparisons of amino acid levels; comparisons were restricted to euthyroid, subclinical hypothyroid, and clinical hypothyroid participants. Following FDR correction, no statistically significant differences in mean serum amino acid levels were observed between the subclinical hypothyroidism, clinical hypothyroidism, and euthyroid groups. Similarly, amino acid concentrations were comparable between the subclinical and clinical hypothyroidism groups (Table 2). However, there were some noteworthy but nonsignificant observations in the mean differences. When comparing the euthyroid and C‐HypoT groups, the mean valine (229.43 ± 58.38 vs. 214.62 ± 54.56), proline (264.64 ± 97.05 vs. 241.55 ± 64.13), and glutamine (419.38 ± 127.62 vs. 404.37 ± 108.95) levels were more different. The mean level of glutamic acid in euthyroid individuals was higher than that in patients with SC‐HypoT (106.36 ± 36.41 vs. 97.74 ± 31.74); although this represented the largest descriptive mean difference, it was not statistically significant after FDR correction.

**TABLE 2:** Serum levels of amino acids according to thyroid disorder subgroups.

Amino acids (μmol/L)	Total (*n* = 810)	Euthyroid (*n* = 658)	Subclinical hypothyroidism (*n* = 122)	Clinical hypothyroidism (*n* = 30)	*p* ^a^ *p* (crude), *p* (FDR)	*p* ^b^ *p* (crude), (FDR)	*p* * *p* (crude), *p* (FDR)
Alanine	449.28 ± 122.73	449.91 ± 124.26	444.56 ± 117.49	452.13 ± 113.16	0.664, 0.949	0.924, 0.988	0.751, 0.987
Aspartic acid	33.65 ± 15.57	34.02 ± 15.67	31.34 ± 14.87	33.17 ± 14.62	0.085, 0.851	0.772, 0.988	0.546, 0.990
Glutamic acid	105.45 ± 36.41	106.36 ± 36.41	97.74 ± 31.74	106.16 ± 39.26	0.016, 0.320	0.977, 0.988	0.219, 0.990
Leucine	119.47 ± 31.70	119.90 ± 31.72	119.34 ± 32.08	114.67 ± 32.76	0.860, 0.983	0.379, 0.988	0.479, 0.990
Methionine	21.42 ± 5.58	21.46 ± 5.56	21.14 ± 5.55	20.51 ± 6.17	0.566, 0.908	0.364, 0.988	0.588, 0.992
Phenylalanine	53.48 ± 16.93	53.68 ± 17.20	51.81 ± 15.37	54.29 ± 17.92	0.271, 0.908	0.849, 0.988	0.477, 0.990
Tyrosine	75.95 ± 19.48	76.05 ± 19.74	76.01 ± 17.53	71.26 ± 19.98	0.983, 0.983	0.195, 0.988	0.199, 0.990
Valine	228.71 ± 57.76	229.43 ± 58.38	229.57 ± 56.33	214.62 ± 54.56	0.978, 0.983	0.174, 0.988	0.193, 0.991
Arginine	66.76 ± 20.41	67.06 ± 20.43	65.47 ± 18.35	60.93 ± 23.01	0.431, 0.908	0.110, 0.988	0.253, 0.990
Citrulline	38.24 ± 12.51	38.12 ± 12.45	38.19 ± 11.93	38.08 ± 13.50	0.955, 0.983	0.988, 0.988	0.965, 0.992
Glycine	245.49 ± 83.05	246.50 ± 79.73	241.22 ± 97.15	236.53 ± 98.31	0.523, 0.908	0.508, 0.508	0.814, 0.992
Ornithine	81.03 ± 25.53	80.61 ± 25.27	82.39 ± 25.82	82.01 ± 31.58	0.458, 0.908	0.771, 0.988	0.945, 0.991
Proline	262.40 ± 95.96	264.64 ± 97.05	252.85 ± 93.43	241.55 ± 64.13	0.223, 0.908	0.198, 0.988	0.532, 0.990
Threonine	199.88 ± 70.33	200.37 ± 71.84	196.22 ± 65.36	190.15 ± 60.54	0.559, 0.908	0.443, 0.988	0.645, 0.992
Serine	122.33 ± 45.32	123.08 ± 46.33	118.92 ± 42.55	121.30 ± 38.59	0.363, 0.908	0.836, 0.988	0.781, 0.990
Histidine	91.92 ± 32.09	92.13 ± 32.75	90.16 ± 31.54	91.11 ± 21.24	0.548, 0.908	0.866, 0.988	0.786, 0.987
Lysine	134.15 ± 63.27	134.63 ± 62.37	133.12 ± 70.72	128.30 ± 49.63	0.812, 0.983	0.548, 0.988	0.726, 0.990
Tryptophane	41.35 ± 13.96	41.58 ± 13.83	41.23 ± 15.20	40.62 ± 13.42	0.806, 0.983	0.710, 0.988	0.841, 0.992
Asparagine	57.74 ± 23.57	57.94 ± 23.42	56.66 ± 25.47	56.31 ± 20.21	0.591, 0.908	0.707, 0.988	0.944, 0.992
Glutamine	417.37 ± 129.02	419.38 ± 127.62	409.08 ± 140.97	404.37 ± 108.95	0.423, 0.908	0.527, 0.988	0.865, 0.987

*Note:* Values are presented as mean ± SD (μmol/L). Group means were compared using independent *t*‐tests with false discovery rate correction.

^a^ Euthyroid versus Subclinical hypothyroidism.

^b^ Euthyroid versus Clinical hypothyroidism.

* Subclinical hypothyroidism versus Clinical hypothyroidism.

Regardless of thyroid hormone status, men had higher mean concentrations of leucine (123.42 vs. 116.77, *p* = 0.015), methionine (22.34 vs. 20.78, *p* = 0.001), and citrulline (40.01 vs. 37.02, *p* = 0.006), whereas women had higher glycine concentrations. (254.23 vs. 232.75, *p* = 0.001). See Table S2.

### 3.3 Association Between Level of Thyroid Hormones and the Level of Amino Acids

In the crude analysis, methionine was nominally inversely associated with TSH (*β* = −0.51, *p* = 0.03). Tyrosine showed a nominal inverse association with TSH in both the crude model (*β* = −0.51, *p* = 0.03) and the adjusted model (*β* = −0.46, *p* = 0.05). Arginine also showed a nominal inverse association with TSH in the adjusted model (*β* = −0.47, *p* = 0.05). However, none of these associations remained statistically significant after correction for multiple comparisons using the FDR procedure. No statistically significant associations were observed between the other amino acids and thyroid biomarkers. (Table 3). Furthermore, Figure 2 illustrates the adjusted correlation patterns between thyroid hormone biomarkers and amino acid concentrations in the whole study population. Overall, the correlation coefficients were generally low, indicating weak correlations between the examined thyroid biomarkers and amino acid levels after adjustment for age, sex, education, BMI, diabetes status, smoking, and levothyroxine use.

**TABLE 3:** Linear regression crude and adjusted *β* coefficients for assessing the association between thyroid hormones and amino acid levels across the whole study population.

Amino acids	TSH		FT4		Anti TPO	
	Crude *β* (95% CI)	Adjusted *β* (95% CI)	Crude *β* (95% CI)	Adjusted *β* (95% CI)	Crude *β* (95% CI)	Adjusted *β* (95% CI)
Alanine	−0.09 (−0.57;0.38) *p* ^a^=0.69, *p* ^&^ = 0.76 *R* ^2^ = 0.001	−0.09 (−0.57;0.40) *p* ^a^=0.72, *p* ^&^ = 0.87 *R* ^2^ = 0.020	0.01 (−0.01;0.03) *p* ^a^=0.23, *p* ^&^ = 0.76 *R* ^2^ = 0.002	0.007 (−0.008;0.02) *p* ^a^=0.35, *p* ^&^ = 0.85 *R* ^2^ = 0.080	1.09 (−6.96;9.14) *p* ^a^=0.79, *p* ^&^ = 0.99 *R* ^2^ = 0.001	−0.26 (−8.35;7.83) *p* ^a^=0.94, *p* ^&^ = 0.99 *R* ^2^ = 0.060
Aspartic acid	−0.23 (−0.71;0.24) *p* ^a^=0.33, *p* ^&^ = 0.56 *R* ^2^ = 0.001	−0.29 (−0.76;0.19) *p* ^a^=0.24, *p* ^&^ = 0.60 *R* ^2^ = 0.030	0.002 (−0.01;0.02) *p* ^a^=0.78, *p* ^&^ = 0.87 *R* ^2^ = 0.001	0.003 (−0.01;0.02) *p* ^a^=0.73, *p* ^&^ = 0.90 *R* ^2^ = 0.070	−2.90 (−10.95;5.15) *p* ^a^=0.48, *p* ^&^ = 0.99 *R* ^2^ = 0.001	−3.64 (−11.61;4.32) *p* ^a^=0.37, *p* ^&^ = 0.74 *R* ^2^ = 0.070
Glutamic acid	−0.25 (−0.72;0.23) *p* ^a^=0.31, *p* ^&^ = 0.56 *R* ^2^ = 0.001	−0.27 (−0.75;0.21) *p* ^a^=0.27, *p* ^&^ = 0.62 *R* ^2^ = 0.030	−0.007 (−0.02;0.01) *p* ^a^=0.37, *p* ^&^ = 0.87 *R* ^2^ = 0.001	−0.007 (−0.02;0.01) *p* ^a^=0.37, *p* ^&^ = 0.85 *R* ^2^ = 0.080	−3.41 (−11.45;4.63) *p* ^a^=0.41, *p* ^&^ = 0.99 *R* ^2^ = 0.001	−4.50 (−12.47;3.47) *p* ^a^=0.27, *p* ^&^ = 0.73 *R* ^2^ = 0.060
Leucine	−0.34(−0.81;0.14) *p* ^a^=0.17, *p* ^&^ = 0.55 *R* ^2^ = 0.002	−0.28 (−0.77;0.21) *p* ^a^=0.25, *p* ^&^ = 0.60 *R* ^2^ = 0.030	0.004 (−0.01;0.02) *p* ^a^=0.62, *p* ^&^ = 0.87 *R* ^2^ = 0.001	0.001 (−0.01;0.02) *p* ^a^=0.87, *p* ^&^ = 0.90 *R* ^2^ = 0.070	−0.05 (−8.11;7.99) *p* ^a^=0.98, *p* ^&^ = 0.99 *R* ^2^ = 0.001	−0.05 (−8.08;7.98) *p* ^a^=0.99, *p* ^&^ = 0.99 *R* ^2^ = 0.050
Methionine	−0.51 (−0.98; −0.03) ***p*** ^**a**^ **=0.03**, *p* ^&^ = 0.45 *R* ^2^ = 0.005	−0.44 (−0.93; −0.04) *p* ^a^=0.07, *p* ^&^ = 0.54 *R* ^2^ = 0.030	0.001 (−0.02;0.02) *p* ^a^=0.89, *p* ^&^ = 0.89 *R* ^2^ = 0.001	0.002 (−0.01;0.02) *p* ^a^=0.79, *p* ^&^ = 0.89 *R* ^2^ = 0.080	0.19 (−7.86;8.25) *p* ^a^=0.96, *p* ^&^ = 0.99 *R* ^2^ = 0.001	0.86 (−7.18;8.89) *p* ^a^=0.83, *p* ^&^ = 0.96 *R* ^2^ = 0.060
Phenylalanine	−0.21 (−0.68;0.26) *p* ^a^=0.40, *p* ^&^ = 0.54 *R* ^2^ = 0.001	−0.25 (−0.73;0.23) *p* ^a^=0.31, *p* ^&^ = 0.64 *R* ^2^ = 0.02	0.004 (−0.01;0.02) *p* ^a^=0.65, *p* ^&^ = 0.87 *R* ^2^ = 0.001	0.005 (−0.01;0.02) *p* ^a^=0.56, *p* ^&^ = 0.89 *R* ^2^ = 0.07	−2.42 (−10.47;5.62) *p* ^a^=0.55, *p* ^&^ = 0.99 *R* ^2^ = 0.001	−3.05 (−10.99;4.89) *p* ^a^=0.45, *p* ^&^ = 0.82 *R* ^2^ = 0.070
Tyrosine	−0.51 (−0.98; −0.04) ***p*** ^**a**^ **=0.03**, *p* ^&^ = 0.44 *R* ^2^ = 0.006	−0.46 (−0.95;0.01) ***p*** ^**a**^ **=0.05**, *p* ^&^ = 0.54 *R* ^2^ = 0.030	0.01 (−0.006;0.03) *p* ^a^=0.23, *p* ^&^ = 0.77 *R* ^2^ = 0.002	0.01 (−0.003;0.03) *p* ^a^=0.11, *p* ^&^ = 0.85 *R* ^2^ = 0.080	1.59 (−6.45;9.64) *p* ^a^=0.69, *p* ^&^ = 0.99 *R* ^2^ = 0.001	1.11 (−6.88;9.08) *p* ^a^=0.78, *p* ^&^ = 0.95 *R* ^2^ = 0.060
Valine	−0.34 (−0.82;0.13) *p* ^a^=0.16, *p* ^&^ = 0.56 *R* ^2^ = 0.003	−0.29 (−0.77;0.21) *p* ^a^=0.24, *p* ^&^ = 0.60 *R* ^2^ = 0.030	−0.002 (−0.02;0.01) *p* ^a^=0.84, *p* ^&^ = 0.87 *R* ^2^ = 0.001	−0.004 (−0.02;0.01) *p* ^a^=0.63, *p* ^&^ = 0.89 *R* ^2^ = 0.070	0.48 (−7.58;8.52) *p* ^a^=0.91, *p* ^&^ = 0.99 *R* ^2^ = 0.001	−0.21 (−8.27;7.86) *p* ^a^=0.96, *p* ^&^ = 0.99 *R* ^2^ = 0.060
Arginine	−0.47 (−0.95;0.07) ***p*** ^**a**^ **=0.05**, *p* ^&^ = 0.51 *R* ^2^ = 0.005	−0.46 (−0.94;0.02) *p* ^a^=0.06, *p* ^&^ = 0.54 *R* ^2^ = 0.040	0.006 (−0.01;0.02) *p* ^a^=0.44, *p* ^&^ = 0.87 *R* ^2^ = 0.001	0.007 (−0.008;0.02) *p* ^a^=0.36, *p* ^&^ = 0.85 *R* ^2^ = 0.090	0.59 (−7.47;8.65) *p* ^a^=0.88, *p* ^&^ = 0.99 R^2^ = 0.001	−0.08 (−8.07;7.92) *p* ^a^=0.98, *p* ^&^ = 0.99 *R* ^2^ = 0.050
Citrulline	0.06 (−0.42;0.53) *p* ^a^=0.82, *p* ^&^ = 0.88 *R* ^2^ = 0.001	0.06 (−0.43;0.55) *p* ^a^=0.81, *p* ^&^ = 0.87 *R* ^2^ = 0.020	−0.01 (−0.03;0.02) *p* ^a^=0.08, *p* ^&^ = 0.77 R^2^ = 0.004	−0.01 (−0.03;0.002) *p* ^a^=0.09, *p* ^&^ = 0.85 *R* ^2^ = 0.080	4.69 (−3.34;12.74) *p* ^a^=0.25, *p* ^&^ = 0.99 R^2^ = 0.002	6.46 (−1.63;14.56) *p* ^a^=0.12, *p* ^&^ = 0.73 *R* ^2^ = 0.070
Glycine	−0.28 (−0.76;0.19) *p* ^a^=0.24, *p* ^&^ = 0.55 *R* ^2^ = 0.002	−0.37 (−0.85;0.12) *p* ^a^=0.13, *p* ^&^ = 0.56 *R* ^2^ = 0.030	−0.002 (−0.02;0.01) *p* ^a^=0.78, p^&^ = 0.87 *R* ^2^ = 0.001	−0.002 (−0.02;0.01) *p* ^a^=0.82, *p* ^&^ = 0.89 *R* ^2^ = 0.080	−2.03 (−10.08;6.02) *p* ^a^=0.62, *p* ^&^ = 0.99 *R* ^2^ = 0.001	−4.42 (−12.44;3.61) *p* ^a^=0.28, *p* ^&^ = 0.85 *R* ^2^ = 0.070
Ornithine	0.20 (−0.28;0.67) *p* ^a^=0.41, *p* ^&^ = 0.53 *R* ^2^ = 0.001	0.18 (−0.30;0.66) *p* ^a^=0.45, *p* ^&^ = 0.67 *R* ^2^ = 0.020	−0.007 (−0.02;0.01) *p* ^a^=0.40, *p* ^&^ = 0.87 *R* ^2^ = 0.001	−0.008 (−0.02;0.007) *p* ^a^=0.29, *p* ^&^ = 0.85 *R* ^2^ = 0.070	6.66 (−1.37;14.71) *p* ^a^=0.11, *p* ^&^ = 0.99 *R* ^2^ = 0.003	5.80 (−2.18;13.78) *p* ^a^=0.15, *p* ^&^ = 0.73 *R* ^2^ = 0.080
Proline	−0.22 (−0.70;0.26) *p* ^a^=0.37, *p* ^&^ = 0.56 *R* ^2^ = 0.001	−0.12 (−0.60;0.36) *p* ^a^=0.62, *p* ^&^ = 0.77 *R* ^2^ = 0.020	0.003 (−0.01;0.02) *p* ^a^=0.72, *p* ^&^ = 0.87 *R* ^2^ = 0.001	0.003 (−0.01;0.02) *p* ^a^=0.70, *p* ^&^ = 0.89 *R* ^2^ = 0.070	−6.17 (−14.21;1.89) *p* ^a^=0.13, *p* ^&^ = 0.99 *R* ^2^ = 0.003	−5.14 (−13.12;2.84) *p* ^a^=0.21, *p* ^&^ = 0.73 *R* ^2^ = 0.070
Threonine	−0.02 (−0.50;0.46) *p* ^a^=0.94, *p* ^&^ = 0.95 *R* ^2^ = 0.001	−0.04 (−0.50;0.44) *p* ^a^=0.87, *p* ^&^ = 0.88 *R* ^2^ = 0.020	−0.01 (−0.03;0.01) *p* ^a^=0.11, *p* ^&^ = 0.76 *R* ^2^ = 0.003	−0.01 (−0.03;0.004) *p* ^a^=0.14, *p* ^&^ = 0.85 *R* ^2^ = 0.080	−2.56 (−10.62;5.43) *p* ^a^=0.53, *p* ^&^ = 0.99 *R* ^2^ = 0.001	−3.84 (−11.79;4.12) *p* ^a^=0.34, *p* ^&^ = 0.73 *R* ^2^ = 0.060
Serine	−0.09 (−0.57;0.38) *p* ^a^=0.69, *p* ^&^ = 0.77 *R* ^2^ = 0.001	−0.15 (−0.63;0.34) *p* ^a^=0.55, *p* ^&^ = 0.85 *R* ^2^ = 0.030	−0.01 (−0.03;0.01) *p* ^a^=0.18, *p* ^&^ = 0.76 *R* ^2^ = 0.005	−0.01 (−0.03;0.006) *p* ^a^=0.21, *p* ^&^ = 0.85 *R* ^2^ = 0.090	−1.84 (−9.89;6.21) *p* ^a^=0.65, *p* ^&^ = 0.99 *R* ^2^ = 0.001	−3.90 (−11.86;4.06) *p* ^a^=0.34, *p* ^&^ = 0.73 *R* ^2^ = 0.070
Histidine	−0.21 (−0.67;0.27) *p* ^a^=0.41, *p* ^&^ = 0.56 *R* ^2^ = 0.001	−0.22 (−0.71;0.26) *p* ^a^=0.36, *p* ^&^ = 0.65 *R* ^2^ = 0.020	−0.01 (−0.03;0.005) *p* ^a^=0.19, *p* ^&^ = 0.76 *R* ^2^ = 0.002	−0.01 (−0.03;0.009) *p* ^a^=0.23, *p* ^&^ = 0.85 *R* ^2^ = 0.08	−4.05 (−12.09;3.99) *p* ^a^=0.32, *p* ^&^ = 0.99 *R* ^2^ = 0.001	−5.21 (−13.15;2.75) *p* ^a^=0.19, *p* ^&^ = 0.73 *R* ^2^ = 0.060
Lysine	−0.27 (−0.75;0.21) *p* ^a^=0.27, *p* ^&^ = 0.56 *R* ^2^ = 0.002	−0.23 (−0.71;0.25) *p* ^a^=0.35, *p* ^&^ = 0.65 *R* ^2^ = 0.030	−0.003 (−0.02;0.01) *p* ^a^=0.73, *p* ^&^ = 0.87 *R* ^2^ = 0.001	−0.001 (−0.02;0.02) *p* ^a^=0.89, *p* ^&^ = 0.89 *R* ^2^ = 0.080	−2.69 (−10.73;5.35) *p* ^a^=0.51, *p* ^&^ = 0.99 *R* ^2^ = 0.001	−1.55 (−9.44;6.35) *p* ^a^=0.70, *p* ^&^ = 0.94 *R* ^2^ = 0.060
Tryptophane	−0.14 (−0.62;0.34) *p* ^a^=0.57, *p* ^&^ = 0.72 *R* ^2^ = 0.005	−0.06 (−0.54;0.42) *p* ^a^=0.81, *p* ^&^ = 0.87 *R* ^2^ = 0.020	−0.002 (−0.02;0.01) *p* ^a^=0.81, *p* ^&^ = 0.87 *R* ^2^ = 0.001	0.001 (−0.01;0.02) *p* ^a^=0.88, *p* ^&^ = 0.89 *R* ^2^ = 0.070	1.27 (−6.78;9.32) *p* ^a^=0.75, *p* ^&^ = 0.99 *R* ^2^ = 0.001	2.45 (−5.55;10.41) *p* ^a^=0.55, *p* ^&^ = 0.83 *R* ^2^ = 0.070
Asparagine	−0.41 (−0.88;0.07) *p* ^a^=0.09, *p* ^&^ = 0.54 *R* ^2^ = 0.004	−0.41 (−0.89;0.07) *p* ^a^=0.09, *p* ^&^ = 0.54 *R* ^2^ = 0.03	0.004 (−0.01;0.02) *p* ^a^=0.61, *p* ^&^ = 0.87 *R* ^2^ = 0.001	0.005 (−0.01;0.02) *p* ^a^=0.55, *p* ^&^ = 0.89 *R* ^2^ = 0.070	−1.03 (−9.09;7.03) *p* ^a^=0.81, *p* ^&^ = 0.99 *R* ^2^ = 0.001	−1.08 (−9.01;6.86) *p* ^a^=0.79, *p* ^&^ = 0.94 *R* ^2^ = 0.050
Glutamine	−0.32 (−0.79;0.16) *p* ^a^=0.18, *p* ^&^ = 0.56 *R* ^2^ = 0.002	−0.28 (−0.76;0.20) *p* ^a^=0.25, *p* ^&^ = 0.60 *R* ^2^ = 0.030	−0.005 (−0.02;0.01) *p* ^a^=0.55, *p* ^&^ = 0.87 *R* ^2^ = 0.001	−0.002 (−0.02;0.01) *p* ^a^=0.75, *p* ^&^ = 0.89 *R* ^2^ = 0.080	−3.33 (−11.37;4.72) *p* ^a^=0.42, *p* ^&^ = 0.99 *R* ^2^ = 0.001	−2.69 (−10.64;5.25) *p* ^a^=0.51, *p* ^&^ = 0.83 *R* ^2^ = 0.070

*Note:* Adjusted for age, sex, education, BMI, diabetes, smoking, levothyroxine use, malnutrition, kidney disease, liver disease, multivitamin use. Statistically significant results (set at 0.05) are shown in bold.

Abbreviations: CI, confidence interval; FT4, free thyroxine; TPO, thyroid peroxidase; TSH, thyroid‐stimulating hormone.

^a^ *p*‐value crude.

^&^ *p*‐value FDR.

**FIGURE 2:**
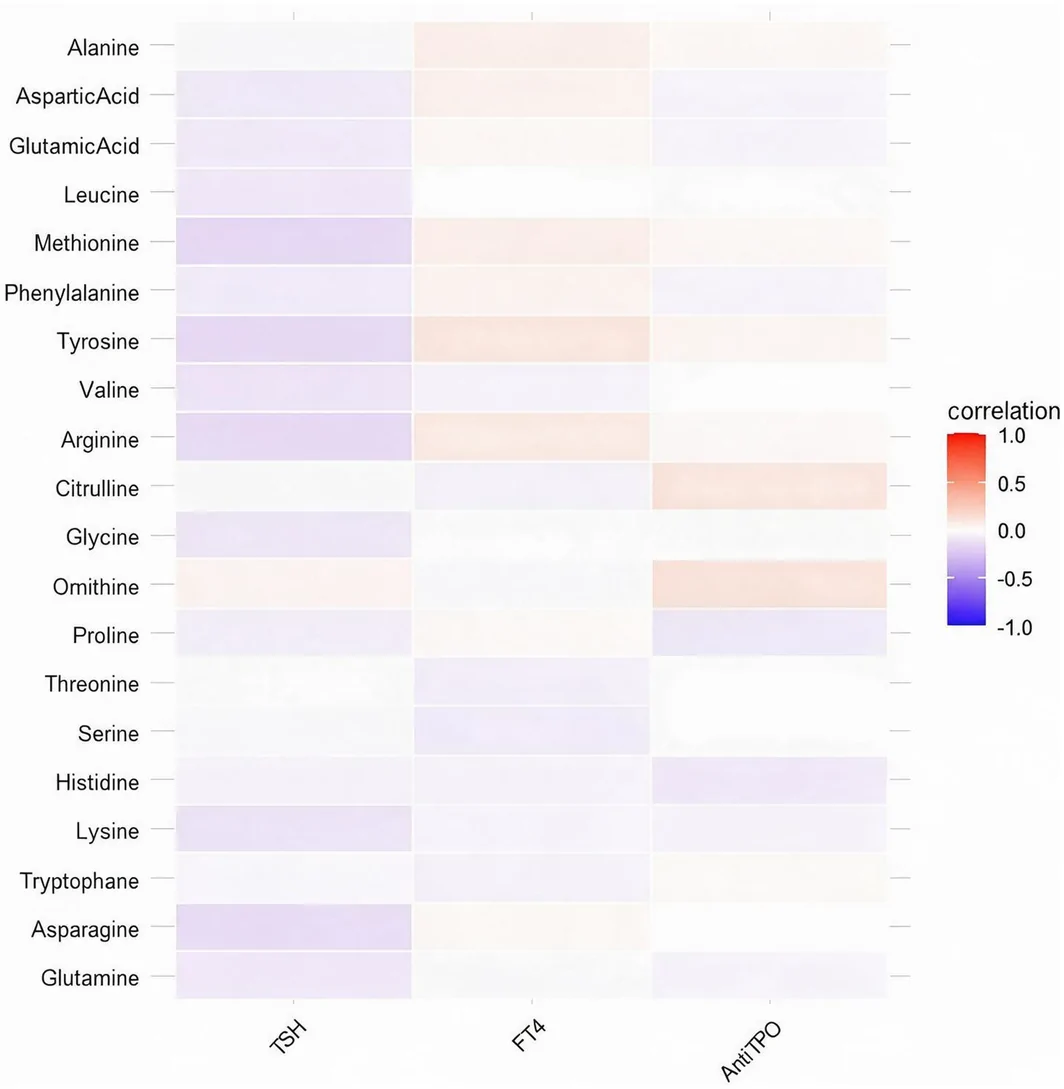
Adjusted correlation of thyroid hormones and amino acid levels in the whole study population, controlling for age, sex, education, BMI, diabetes status, smoking, and levothyroxine use. Colour scale indicates correlation coefficient magnitude and direction (−1–1). TSH, Thyroid‐stimulating hormone; FT4, Free thyroxine; TPO, Thyroid peroxidase; CI, Confidence interval.

## 4 Discussion

In this targeted metabolomics study, we examined the associations between circulating amino acid concentrations and thyroid function biomarkers in adults aged 50 years and older participating in the Bushehr Elderly Health Program. Among the 824 participants initially enrolled, 14 individuals with clinical or subclinical hyperthyroidism were excluded from the primary analytical comparisons because of the very small subgroup sizes. The main analyses therefore included 810 participants with euthyroid status, subclinical hypothyroidism, or clinical hypothyroidism. No statistically significant differences in mean aminoacid concentrations were observed between the thyroid disorder groups after FDR correction. Although several nominal associations were observed between selected amino acids and TSH in crude or covariate‐adjusted analyses, none remained statistically significant after correction for multiple comparisons. Descriptive differences in the mean concentrations of selected amino acids were also considered exploratory and should not be interpreted as confirmed metabolic alterations. Beyond the primary objectives of this study, we identified sex‐specific differences in the circulating levels of several amino acids. Specifically, men had higher mean concentrations of leucine, methionine, and citrulline, while women demonstrated higher mean glycine concentrations.

Measuring serum levels of TSH and FT4 is currently the primary method for diagnosing conditions like clinical and subclinical hypothyroidism and hyperthyroidism [38]. However, the potential metabolic implications of these conditions have recently increased interest in characterizing their underlying metabolic alterations, particularly in subclinical forms that often occur without overt signs or symptoms but may still contribute to long‐term adverse outcomes [39, 40]. Several studies have reported alterations in amino acid profiles in thyroid disorders. A recent analysis demonstrated decreased levels of tyrosine, lysine, and histidine, along with increased glycine concentrations, in patients with clinical hypothyroidism (C‐HypoT) [23]. A nuclear magnetic resonance (NMR)‐based metabolomics study of patients with C‐HypoT by Piras et al. observed reduced proline levels and increased serine concentrations [22]. Furthermore, experimental evidence from animal models has demonstrated that hypothyroidism may impair glutamine metabolism, as reflected by reduced plasma and skeletal muscle glutamine levels and decreased glutamine release from skeletal muscle [41]. Unlike some previous studies, we found no statistically significant differences in mean serum amino acid concentrations between participants with clinical or subclinical hypothyroidism and euthyroid participants after FDR correction. Although lower descriptive mean levels of valine, proline, glutamine, and glutamic acid were observed in selected comparisons, these findings were exploratory and did not remain statistically significant after correction for multiple comparisons. Therefore, they should not be interpreted as evidence of thyroid‐related alterations in these amino acids. The existing literature shows considerable heterogeneity regarding serum amino acid alterations in thyroid disorders. While some studies have reported increased concentrations of amino acids such as lysine, glutamine, arginine, alanine, asparagine, methionine, and aspartic acid in individuals with impaired thyroid function, others have described contrasting patterns. These discrepancies emphasize the complexity of amino acid metabolism in thyroid disorders and the need to further investigate the potential influence of population characteristics, disease status, analytical methods, and other metabolic factors [25, 29, 42, 43, 44].

The observed discrepancies across studies likely reflect methodological heterogeneity rather than random variation, including differences in analytical platforms, fasting versus non‐fasting sampling, the timing of hormone measurements, sample size, and participant characteristics. Analytical platforms vary: Piras et al. and Janovsky et al. used NMR‐based approaches, generally less sensitive for low‐abundance amino acids than the targeted FI‐MS/MS panel used here, while Shao et al. used untargeted metabolomics without amino acid‐specific quantification. Participant characteristics also diverge. Janovsky et al. studied a younger, broader cohort (mean age 51.3 ± 9.0, *n* = 4098) limited to branched‐chain amino acids and alanine, while Piras et al. examined a small (*n* = 18), longitudinal pre−/post‐treatment sample of overt hypothyroid patients rather than a cross‐sectional spectrum of disease severity. Disease severity and medication handling differed as well: Janovsky et al. excluded levothyroxine‐treated participants entirely, whereas we retained and statistically adjusted for this exposure. Fasting protocols ranged from a fixed 12‐h fast (Janovsky et al.) to the 8–12‐h window used here, and statistical approaches spanned simple correlation, machine‐learning classification, and FDR‐corrected regression, further complicating direct comparison across studies [22, 23, 25]. Notably, our study focused on individuals aged 50 years and older. Aging is accompanied by multiple physiological changes that substantially influence human metabolism. Age‐related alterations, including changes in hormonal regulation, reductions in muscle mass and lean body mass, impaired hepatic and renal function, and declines in basal metabolic rate (BMR) and resting metabolic rate (RMR), may contribute to disturbances in protein and amino acid metabolism [45, 46, 47, 48, 49]. Furthermore, decreased gastric acid and digestive enzyme secretion, impaired intestinal motility, and reduced activity of amino acid transporter proteins in the intestine and skeletal muscle may affect protein digestion, amino acid absorption, and amino acid availability, thereby altering amino acid homeostasis in older adults [50, 51, 52]. Therefore, age likely contributes to the observed variations in amino acid profiles, underscoring the complexity of interpreting metabolomics data in older populations.

Among the amino acids demonstrating the largest mean differences in our study, valine, proline, glutamine, and glutamic acid may represent potential metabolic markers associated with thyroid dysfunction. Thyroid hormones are involved in the regulation of glutamine metabolism through the induction of mitochondrial glutamate pyruvate transaminase 2 (GPT2), which supports glutamine‐dependent anaplerotic flux and skeletal muscle maintenance [53]. Additionally, glutamine synthesis could be reduced following several conditions, including thyroid dysfunction [41, 54]. The descriptively lower proline levels may be compatible with alterations in protein synthesis or collagen metabolism; however, this interpretation remains speculative [55, 56, 57]. The descriptively lower valine levels are compatible with previous reports concerning branched‐chain amino acid (BCAA) metabolism; however, this observation was exploratory [25].

In the present study, several nominal associations were observed between amino acid concentrations and thyroid biomarkers before correction for multiple comparisons. However, none of these associations remained statistically significant after FDR correction. Tyrosine and arginine showed nominal inverse associations with TSH in crude or adjusted models, but the findings should be interpreted cautiously and considered hypothesis‐generating. Because the cross‐sectional design cannot establish temporality, and because the associations did not remain significant after correction for multiple testing, these results do not provide evidence of a confirmed or clinically meaningful relationship between tyrosine, arginine, and thyroid function. Tyrosine, a non‐essential amino acid derived from phenylalanine, serves as a critical substrate for thyroid hormone synthesis, particularly thyroxine production [58]. Beyond its role in thyroid hormone biosynthesis, tyrosine is involved in intracellular signalling pathways through phosphorylation by tyrosine kinases, which regulate a wide range of biological processes, such as cellular growth, differentiation, and metabolic regulation [59, 60]. Tyrosine has established roles in thyroid hormone synthesis and cellular signalling, which may provide a biological context for the nominal association observed in the uncorrected analyses [61, 62]. However, because this association did not remain statistically significant after FDR correction, the finding should be considered exploratory and does not establish a thyroid‐related alteration in tyrosine metabolism. Experimental studies have suggested that thyroid hormones upregulate L‐arginine transporters and enhance arginine uptake in endothelial cells [63]; however, this mechanism cannot be considered an explanation for the present findings because the observed association between arginine and TSH did not remain statistically significant after FDR correction. Further longitudinal and experimental studies are required to evaluate whether this relationship is reproducible.

Previous metabolomic research has shown differences in serum amino acid levels based on sex. Men usually have higher levels of branched‐chain amino acids, such as leucine, and other essential amino acids [64, 65, 66, 67, 68]. Our findings are consistent with those of previous studies, which reported higher leucine and methionine concentrations in men. Differences in muscle metabolism and muscle mass, together with higher dietary protein intake among men, may partly explain this finding [69]. Consistent with our results, several studies have reported higher glycine concentrations in women compared with men. Proposed explanations for this sex‐related difference include variations in metabolic regulation, hormonal influences, and differences in amino acid utilization and turnover [70, 71, 72]. The lack of significant differences in amino acid concentrations between thyroid disorder groups in our study, along with inconsistencies observed in previous investigations, suggests that the interplay between amino acid metabolism and thyroid function is far more complex than anticipated. These heterogeneous and sometimes conflicting findings emphasize the need for further research to clarify the mechanisms underlying these associations and to determine their potential clinical relevance.

A key strength of this study is its relatively large initial sample of 824 participants, with primary analyses conducted in 810 participants after exclusion of the very small hyperthyroid subgroups, which enhances statistical power. The study also adjusted for key demographic and clinical confounders, including age, sex, BMI, diabetes, smoking status, levothyroxine use, malnutrition status, kidney disease, liver disease, and multivitamin use, which improves the reliability of the estimates. However, this study has several limitations. Although identifying associations is an important objective in metabolomics research, the cross‐sectional design limits the ability to establish causal relationships. Furthermore, the inclusion of individuals aged 50 years and older may limit the generalizability of our findings to younger populations or other age groups. Additionally, as this study was conducted in Bushehr, Iran, the findings may be influenced by regional and population‐specific characteristics, which could affect their applicability to other ethnic or geographic populations. Although blood samples were collected under a standardized morning fasting protocol, the permitted 8–12‐h fasting window may have introduced some variability in circulating amino acid concentrations. The marked imbalance in subgroup sizes, particularly for clinical and subclinical hyperthyroidism, limited our ability to detect clinically meaningful differences in amino acid levels across thyroid disorder categories. While excluding these groups from between‐group comparisons was necessary to avoid unstable estimates from very small cells, this means our findings on categorical thyroid disorder status are most applicable to euthyroid and hypothyroid populations and cannot be generalized to hyperthyroidism. The very small number of participants with overt thyroid disease raises the possibility of a Type II error, whereby true differences between groups may have gone undetected due to insufficient statistical power. Also, our targeted amino acid analysis limits our ability to examine a broader range of metabolites and conduct pathway analysis. In addition, isoleucine and cysteine were not available in the measured amino acid dataset and therefore could not be included in the analysis, which may have limited the comprehensiveness of the amino acid assessment. Furthermore, the analytical distinction between leucine and isoleucine may be challenging depending on chromatographic resolution and should be considered when interpreting leucine‐related findings.

Lastly, unmeasured factors, including alcohol consumption, physical activity, medication use other than levothyroxine, inflammatory status, and other endocrine disorders, might have affected the results.

## 5 Conclusion

In this study, no statistically significant differences in amino acid concentrations were observed between euthyroid participants and those with subclinical or clinical hypothyroidism after FDR correction. Although several nominal associations were observed between selected amino acids and TSH in crude or adjusted analyses, none remained statistically significant after correction for multiple comparisons. These findings do not support clinically meaningful alterations in amino acid profiles or robust associations between the examined amino acids and thyroid biomarkers. Larger longitudinal studies with broader metabolomic coverage and prespecified hypotheses are needed to determine whether the nominal findings observed in this study are reproducible and biologically meaningful.

## Author Contributions

**Mohamad Mehdi Khadembashiri:** writing – original draft, writing – review and editing. **Kazem Khalagi:** methodology, validation, writing – review and editing. **Shahrzad Mohseni:** investigation, writing – review and editing. **Alireza Amanollahi:** formal analysis, writing – review and editing. **Fatemeh Bandarian:** data curation, writing – review and editing. **Noushin Fahimfar:** supervision, data curation, validation, writing – review and editing. **Iraj Nabipour:** project administration. **Mahnaz Pejman Sani:** conceptualization, supervision, validation, writing – review and editing. **Farideh Razi:** conceptualization, data curation, validation, writing – review and editing.

## Funding

The authors have nothing to report.

## Ethics Statement

This cross‐sectional study was performed after the institutional board review and approval by ethics committee of Tehran University of medical sciences (Ethics Code: IR.TUMS.EMRI.REC.1403.034). Written informed consent was obtained from all the participants. All research procedures were conducted in accordance with the Declaration of Helsinki and its later amendments.

## Consent

Written informed consent was obtained from all enrolled participants.

## Conflicts of Interest

The authors declare no conflicts of interest.

## Supporting information


**Table S1:** Baseline characteristics of participants based on gender.
**Table S2:** Serum levels of metabolites according to gender.

## Data Availability

The data that support the findings of this study are available from the corresponding author upon reasonable request.
