# Supplementary material for: Association Between Amino Acid Levels and Thyroid Function Among Iranian Adults: A Cross‐Sectional Analysis of Bushehr Elderly Health (BEH) Program
Source: Endocrinol Diabetes Metab. 2026 Nov 3;9(6):e70375. doi: 10.1002/edm2.70375 (PMC13731737; doi:10.1002/edm2.70375)
Supplement: Supplementary file 1 [file EDM2-9-e70375-s001.docx]

**Association between Amino Acid Levels and Thyroid Function among Iranian Adults: A Cross-Sectional Analysis of Bushehr Elderly Health (BEH) Program**

**Correspondence**

^≠^Mahnaz Pejman Sani, Endocrinology and Metabolism Research Center, Endocrinology and Metabolism Clinical Sciences Institute, Tehran University of Medical Sciences, Tehran, Iran.

E-Mail: [mpsani@sina.tums.ac.ir](mailto:mpsani@sina.tums.ac.ir)

**Supplementary Table 1** Baseline characteristics of participants based on gender

| **Parameters** | **Total (n=824)** | **Men (n=335)** | **Women (n=489)** | **p-value** |
| --- | --- | --- | --- | --- |
| **Age [Mean ± Standard Deviation]** | 61.79± 7.96 | 62.27± 8.57 | 61.47± 7.52 | 0.158 |
| **BMI [Mean ± Standard Deviation]** | 28.30± 5.01 | 26.52± 4.18 | 29.52± 5.16 | **<0.001** |
| **Smoking [No. (%)]**  Yes  No | 188 (22.8)  636 (77.2) | 87 (26.0)  248 (74.0) | 101 (20.7)  388 (79.3) | 0.074 |
| **Education [No. (%)]**  Illiterate  Primary school  Secondary school  Academic | 96 (11.7)  268 (32.5)  270 (32.8)  190 (23.1) | 67 (20.0)  122 (36.4)  102 (30.4)  44 (13.1) | 29 (5.9)  146 (29.9)  168 (34.4)  146 (29.9) | **<0.001** |
| **Diabetes mellitus [No. (%)]**  Yes  No | 293 (35.8)  526 (64.2) | 107 (32.2)  225 (67.8) | 186 (38.2)  301 (61.8) | 0.081 |
| **Levothyroxine [No. (%)]**  Yes  No | 118 (14.3)  706 (85.7) | 17 (5.1)  318 (94.9) | 101 (20.7)  388 (79.3) | **<0.001** |
| **SBP [Mean ± Standard Deviation]** | 131.11± 19.76 | 134.33± 20.08 | 128.89± 19.20 | **<0.001** |
| **DBP [Mean ± Standard Deviation]** | 80.25± 10.52 | 81.93± 10.67 | 79.09± 10.26 | **<0.001** |
| **TSH (mIU/mL) [Mean ± Standard Deviation]** | 3.65± 6.77 | 3.29± 5.53 | 3.89± 7.49 | 0.205 |
| **FT4 (ng/dl) [Mean ± Standard Deviation]** | 1.26± 0.25 | 1.26± 0.24 | 1.27± 0.27 | 0.664 |
| **Anti TPO (IU/ml) [Mean ± Standard Deviation]** | 66.34± 117.43 | 53.97± 101.13 | 74.81±126.81 | **0.009** |
| **Cholesterol (mg/dL)** **[Mean ± Standard Deviation]** | 189.63± 42.59 | 182.07± 37.59 | 194.83± 45.01 | **<0.001** |
| **LDL-C (mg/dL) [Mean ± Standard Deviation]** | 110.08± 37.17 | 106.04± 33.51 | 112.86± 39.28 | **0.008** |
| **HDL-C (mg/dL)** **[Mean ± Standard Deviation]** | 48.79± 11.19 | 44.86± 10.35 | 51.49± 10.95 | **<0.001** |
| **Triglyceride (mg/dL)** **[Mean ± Standard Deviation]** | 152.68± 87.55 | 155.83± 94.77 | 150.52± 81.90 | 0.405 |
| **Dyslipidemia [No. (%)]**  Yes  No | 602 (73.1)  222 (26.9) | 228 (68.1)  107 (31.9) | 374 (76.5)  115 (23.5) | **0.007** |
| **Urea (mg/dL) [Mean ± Standard Deviation]** | 31.28± 10.46 | 33.78± 12.16 | 29.57± 8.73 | **<0.001** |
| **Cr (mg/dL) [Mean ± Standard Deviation]** | 1.07± 0.35 | 1.22± 0.32 | 0.97± 0.33 | **<0.001** |
| **ALT (IU/L) [Mean ± Standard Deviation]** | 19.48± 9.69 | 20.72± 10.29 | 18.63± 9.18 | **0.002** |
| **AST (IU/L) [Mean ± Standard Deviation]** | 21.52± 7.63 | 22.40± 8.98 | 20.91± 6.49 | **0.010** |
| **ALP (IU/L) [Mean ± Standard Deviation]** | 227.48± 62.14 | 217.48± 62.14 | 234.59± 64.96 | **<0.001** |
| Abbreviations: BMI- Body mass index; SBP-Systolic blood pressure; DBP-Diastolic blood pressure; TSH- Thyroid stimulating hormone; FT4-Free thyroxine; TPO-Thyroid peroxidase; LDL-C- Low density lipoprotein-cholesterol; HDL-C- High density lipoprotein-cholesterol; Cr- Creatinine; ALT- Alanine transaminase; AST- Aspartate aminotransferase; ALP- Alkaline phosphatase | | | | |

**Supplementary Table 2** Serum levels of metabolites according to gender

| **Amino Acids (µmol/L)** | **Men (n=332)** | **Women (n=478)** | **p-value** |
| --- | --- | --- | --- |
| **Alanine** | 446.43± 121.72 | 451.23± 123.49 | 0.779 |
| **Aspartic Acid** | 32.69± 14.32 | 34.30± 16.26 | 0.272 |
| **Glutamic Acid** | 105.30± 35.52 | 105.54± 37.04 | 0.963 |
| **Leucine** | 123.42± 32.58 | 116.77± 30.82 | **0.015*** |
| **Methionine** | 22.34± 5.62 | 20.78± 5.46 | **0.001*** |
| **Phenylalanine** | 53.02± 15.92 | 53.79± 17.60 | 0.746 |
| **Tyrosine** | 76.91± 19.74 | 75.29± 19.29 | 0.449 |
| **Valine** | 232.87± 58.10 | 225.84± 57.40 | 0.251 |
| **Arginine** | 66.67± 18.47 | 66.82± 21.65 | 0.963 |
| **Citrulline** | 40.01± 12.58 | 37.02± 12.33 | 0.006 |
| **Glycine** | 232.75± 67.85 | 254.23± 91.05 | **0.001*** |
| **Ornithine** | 81.92± 24.32 | 80.41± 26.33 | 0.625 |
| **Proline** | 269.04± 96.16 | 257.84± 95.66 | 0.255 |
| **Threonine** | 204.42± 70.11 | 196.76± 70.38 | 0.272 |
| **Serine** | 118.77± 40.37 | 124.76± 48.32 | 0.191 |
| **Histidine** | 91.74± 31.67 | 92.05± 32.40 | 0.963 |
| **Lysine** | 134.48± 66.92 | 133.91± 60.70 | 0.962 |
| **Tryptophane** | 42.47± 14.45 | 40.59± 13.59 | 0.193 |
| **Asparagine** | 58.72± 24.23 | 57.07± 23.10 | 0.543 |
| **Glutamine** | 417.12± 132.16 | 417.55± 126.94 | 0.963 |
